# Nesfatin-1 protects H9c2 cardiomyocytes against cobalt chloride-induced hypoxic injury by modulating the MAPK and Notch1 signaling pathways

**DOI:** 10.1186/s40709-021-00147-4

**Published:** 2021-09-13

**Authors:** Mingchen Li, Kai Li, Yuan Ren

**Affiliations:** 1 Department of Cardiology, The People’s Hospital of Jiulongpo, No. 31 Baixin Road, Baishiyi, Jiulongpo, 401329 Chongqing, People’s Republic of China; 2 Heart Center, The First Affiliated Hospital of Xinjiang Medical University, Xinjiang 830054 Urumqi, People’s Republic of China

**Keywords:** CoCl_2_, Nesfatin-1, ROS, MAPK, Notch1

## Abstract

**Background:**

This study aimed to explore the effect of nesfatin-1 on cobalt chloride (CoCl_2_)-induced hypoxic injury in cardiomyocyte H9c2 cells.

**Methods:**

H9c2 cardiomyocytes were induced by different concentrations of CoCl_2_ to mimic the hypoxia condition. Cell viability was detected by MTT assay. Cell apoptosis was detected by TUNEL staining and flow cytometry. ROS production was detected using the fluorescence probe DCFH-DA. The mitochondrial membrane potential (MMP) was detected using the TMRE method. The levels of released lactate dehydrogenase (LDH), malondialdehyde (MDA), superoxide dismutase (SOD), glutathione (GSH), and catalase (CAT) were detected using the commercial kits. The protein levels of MAPK signaling members (p-JNK1/2, p-ERK1/2, and p-p38) and Notch1 signaling members (Notch1, Hes 1, and Jagged 1) were detected by Western blot.

**Results:**

CoCl_2_ significantly promoted cell apoptosis, increased LDH leakage, MDA concentration, and decreased cell viability, SOD activity, GSH production, and CAT activity. CoCl_2_-induced hypoxic injury in H9c2 cells was partially restored by nesfatin-1 treatment. Moreover, nesfatin-1 treatment attenuated CoCl_2_-induced increase in ROS production and mitochondrial dysfunction, decreased mitochondrial membrane potential, Bax/Bcl-2 imbalance, as well as c-caspase-9 and c-caspase-3 levels. Moreover, nesfatin-1 treatment inhibited the activation of MAPK and Notch1 signaling pathways.

**Conclusions:**

Nesfatin-1 could effectively protect H9c2 cells against CoCl_2_-induced hypoxic injury by blocking MAPK and Notch1 signaling pathways, suggesting that nesfatin-1 might be a promising therapeutic agent for hypoxic cardiac injury.

## Background

In the past decades, numerous studies have demonstrated that hypoxia-induced dysfunction is one of the most important components of numerous diseases [1]. Tissue hypoxia is often caused by oxygen partial pressure in arterial blood and disruptive blood flow [2]. Previous studies have reported that the imbalance between O_2_ supply and the heart’s demand may cause distant organ damages [3]. Hypoxia occurred in hearts may lead to cardiomyocyte injury, which is involved in various serious heart diseases such as myocardial infarction and ischemic stroke [4, 5]. Therefore, inhibition of hypoxia-induced cardiomyocyte damages is a promising therapeutic strategy for hypoxic cardiac injury.

In a hypoxic state, mitochondria may be a threat to cells due to the production of reactive oxygen species (ROS) [6]. The excess ROS may result in cardiomyocyte damages, including apoptosis which is characterized by the increased release of lactate dehydrogenase (LDH) and malondialdehyde (MDA) and decreased superoxide dismutase (SOD), glutathione (GSH), and catalase (CAT) [7]. The mitogen-activated protein kinase (MAPK) family is highly conserved and known to respond to various stresses via transmitting the signals from cytoplasmic to nuclear targets, which is mediated by a phosphorylation cascade [8]. MAPK signaling pathway has been identified to be associated with cardiomyocyte apoptosis triggered by ROS [9]. Another signaling pathway triggered by ROS is the Notch pathway, and activation of the Notch pathway can induce downstream proteins involved in the cell cycle and apoptosis, including Notch1, Hes-1, Hes-5, and Jagged1 [10, 11]. These reports indicate that targeting MAPK and Notch1 signaling pathways may be a potential treatment to prevent ROS-induced cardiomyocyte injury.

Nesfatin-1 is an 82 amino acid polypeptide derived from the nucleobindin 2 (NUCB2) protein [12]. Increasing evidence has revealed its wide functions in human diseases. For example, nesfatin-1 is identified earliest to participate in the regulation of food intake by acting as an anorexigenic peptide to cause weight loss and malnutrition [13]. Nesfatin-1 exerts cardiovascular protective effects by inhibiting peripheral arterial remodeling [14]. In addition, nesfatin-1 also plays important roles in different types of cancers, such as gastric cancer [15], bladder cancer [16], colon cancer [17], ovarian epithelial carcinoma [18], and so on. Recently, Nazarnezhad et al. demonstrated that nesfatin-1 could significantly reduce high glucose-induced intracellular ROS production in PC12 cells in diabetic neuropathy [19], suggesting that nesfatin-1 might have potential roles in hypoxia-induced cell injury.

Cobalt chloride (CoCl_2_) is widely used to mimic hypoxia conditions [20]. This study found that CoCl_2_ significantly promoted cell apoptosis and decreased cell viability while increased ROS production and caused mitochondrial abnormalities. Meanwhile, the expression of MAPK signaling members (p-JNK1/2, p-ERK1/2, and p-p38) and Notch1 signaling members (Notch1, Hes 1, and Jagged 1) was evaluated by CoCl_2_ treatment. Moreover, our study revealed that nesfatin-1 effectively attenuated CoCl_2_-induced H9c2 cell injury by inhibiting MAPK and Notch1 signaling pathways, suggesting that nesfatin-1 might be an effective agent against cardiac hypoxia.

## Methods

### Cell culture

Rat embryonic cardiomyocyte cell line H9c2 was obtained from ATCC (Rockville, USA) and cultured in DMEM containing 10 % FBS (Gibco, Carlsbad, CA, USA) at 37 °C and additional 5% CO_2_.

### MTT assay

Cell viability was measured using the MTT assay kit (ab211091, Abcam, USA). In brief, approximately 5 × 10^4^ cells were placed into each well of 96-well plates and cultured overnight. To evaluate the effect of CoCl_2_, cells were treated with CoCl_2_ at different concentrations (400, 500, 600, 800, 1000, and 1200 µM) for 24 or 48 h. To evaluate the effect of nesfatin-1, cells were treated with nesfatin-1 at different concentrations (10, 20, 40, 60, 80, 100, and 120 nM) after incubation with 800 µM CoCl_2_ for 48 h for another 24 h. Subsequently, 5 mg ml^− 1^ MTT reagent was added to each well, and the plates were incubated for 4 h. The absorption at 570 nm was detected with an ELISA plate reader.

### TUNEL staining assay

The apoptotic cells were visualized using TUNEL Apoptosis Detection Kit (Yeasen, Shanghai, China). Approximately, 1 × 10^5^ cells per well were seeded into 24-well plates and treated with 800 µM CoCl_2_ for 48 h before treated with nesfatin-1 at different concentrations (20, 60, and 80 nM) for another 24 h. Then cells were incubated with Alexa Fluor 488-12-dUTP Labeling Mix for 20 min, and the nuclei were counterstained by DAPI solution. The apoptotic cells were counted under a fluorescence microscope (Olympus, Tokyo, Japan) (× 40).

### Annexin V apoptosis assay

Cell apoptosis was detected using the Annexin V-APC and 7-AAD Apoptosis Detection reagent (BD Biosciences) as previously described [21]. Briefly, 2 × 10^5^ cells per well were plated into 6-well plates and treated with 800 µM CoCl_2_ for 48 h before treatment with nesfatin-1 at different concentrations (20, 60, and 80 nM) for another 24 h. Afterward, cells were washed and incubated Annexin V-conjugated APC and 7-AAD. Cell apoptosis was detected using a flow cytometer (Beckman, Pasadena, USA).

### Western blot

Total proteins were extracted from the cultured cells using RIPA lysis buffer containing 1% NP-40, 0.1% SDS, 0.5% sodium deoxycholate, 50 mM Tris pH 7.5, and 150 mM NaCl (Beyotime Institute of Biotechnology, Shanghai, China). Approximately equal amounts of protein samples (25 µg) were separated by 12% SDS-PAGE and transferred onto PVDF membranes. After blocking in 5% nonfat milk, the membranes were incubated overnight at 4 °C with primary antibodies against p38 (ab31828, 1:1000, Abcam, USA), p-p38 (ab195049, 1:1000, Abcam, USA), ERK1/2 (ab184699, 1:1000, Abcam, USA), p-ERK1/2 (ab223500, 1:1000, Abcam, USA), JNK1/2 (#9255, 1:1000, Cell Signaling Technology, USA), p-JNK1/2 (#4668, 1:1000, Cell Signaling Technology, USA), Notch1 (#3608, 1:1000, Cell Signaling Technology, USA), Hes 1 (#11,988, 1:1000, Cell Signaling Technology, USA), Jagged 1 (#70109, 1:1000, Cell Signaling Technology, USA), Bax (ab32503, 1:1000, Abcam, USA), Bcl2 (ab32124, 1:1000, Abcam, USA), c-caspase-9 (ab2324, 1:1000, Abcam, USA), c-caspase-3 (ab32042, 1:1000, Abcam, USA) and β-actin (ab8226, 1:10,000, Abcam, USA). On the next day, the membranes were then incubated with HRP-conjugated secondary antibody for 2 h. The antibody-bound proteins were detected using an ECL chemiluminescence detection kit (Thermo Fisher Scientific, Waltham, MA, USA).

### Measurement of intracellular ROS

The intracellular ROS in H9c2 cells after different treatments was detected using 2′,7′-dichlorodihydrofluorescein diacetate (DCFH-DA) probe (Sigma Chemicals Co., USA) as previously reported [22]. Briefly, 5 × 10^5^ cells per well were seeded into 24-well plates and treated with 800 µM CoCl_2_ for 48 h, followed by 20, 60, and 80 nM nesfatin-1 for 24 h. After addition of 25 µM DCF-DA solution, the plates were incubated for 30 min, and the fluorescence of DCF was detected using a microplate reader (Bio-Rad, USA).

### Detection of LDH, MDA, SOD, GSH, and CAT

Approximately 2 × 10^5^ cells per well were plated into 24-well plates and treated with 800 µM CoCl_2_ for 48 h, followed by 20, 60, and 80 nM nesfatin-1 for 24 h. The levels of released LDH, MDA, SOD, GSH, and CAT in the supernatant were detected using specific detection kits.

### Detection of mitochondrial membrane potential (MMP)

The MMP was detected as previously described [23]. In brief, 5 × 10^5^ cells per well were plated into 6-well plates and treated with 800 µM CoCl_2_ for 48 h, followed by 20, 60, and 80 nM nesfatin-1 for 24 h. The cells were incubated with 2.5 nM tetramethylrhodamine ethyl ester (Sigma-Aldrich, St Louis, MO, USA; Merck KGaA, Darmstadt, Germany) for 25 min. After cells were washed with PBS twice, the fluorescence at 490 nm (excitation)/590nm (emission) was detected using a flow cytometer (Beckman, Pasadena, USA).

### Statistical analysis

The data were presented as mean  ±  SD (standard deviation). Data analysis was performed in SPSS ver. 22.0 (IBM Corp, Armonk, NY, USA). The difference between two groups was tested using Student’s *t *test . *p * <  0.05 was considered significant.

## Results

### CoCl_2_ inhibited proliferation, induced apoptosis, and activated MAPK and Notch1 signaling pathways in H9c2 cells

**Fig. 1:**
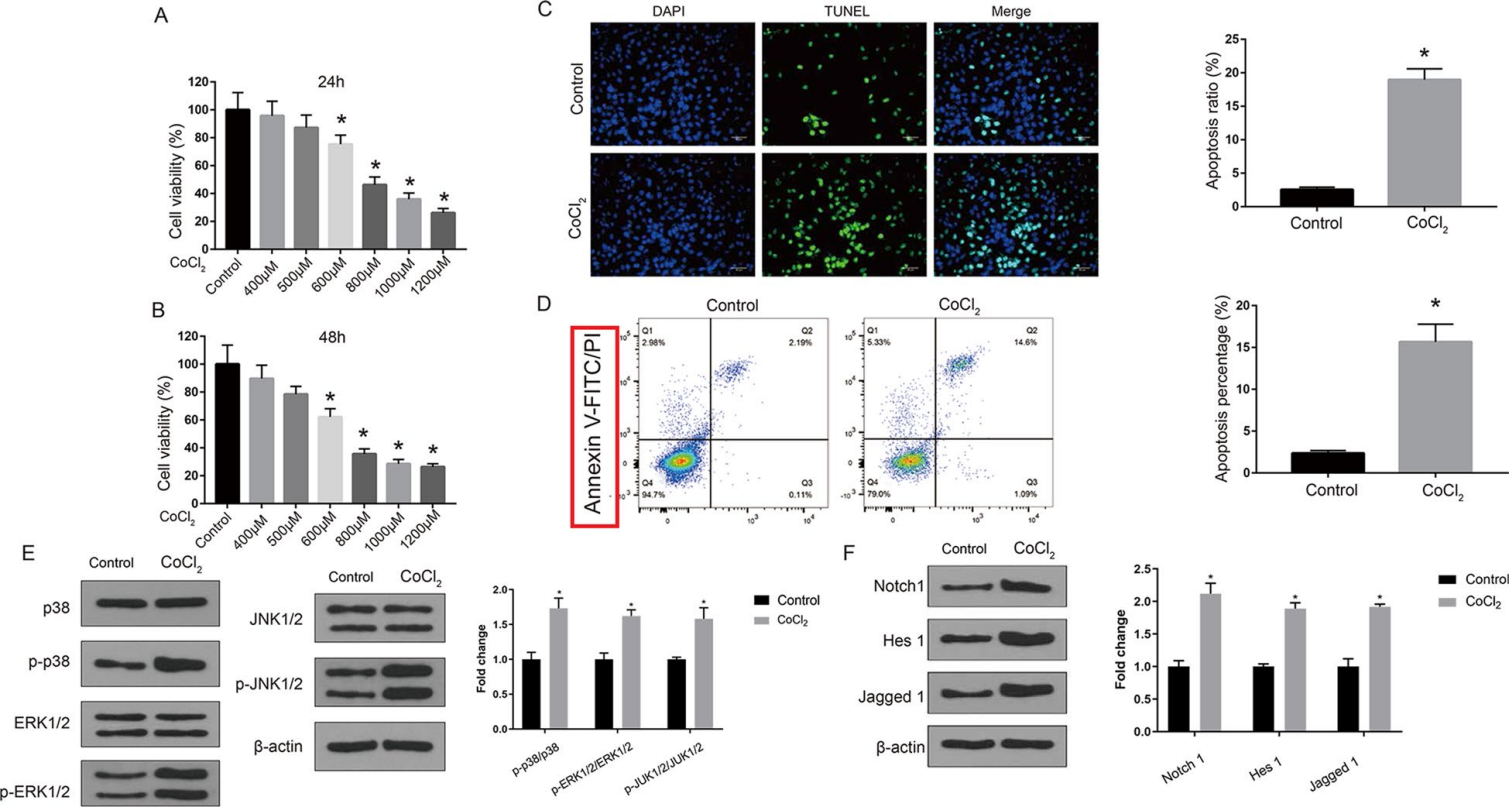
Cobalt chloride inhibited proliferation and induced apoptosis in H9c2 cells. **A**, **B** H9c2 cells were treated with CoCl_2_ (400, 500, 600, 800, 1000, and 1200 µM) for 24 (**A**) or 48 h (**B**). Cell viability was detected by MTT assay. **C**–**F** H9c2 cells were treated with 800 µM CoCl_2_ for 48 h. Cell apoptosis was detected by TUNEL staining assay (**C**) and flow cytometry (**D**). The expression of MAPK pathway members (**E**) and Notch1 pathway members (**F**) was detected by Western blot. Data are presented as mean  ±  SD. *p  <  0.05 vs. control group (0 µM CoCl_2_)

To confirm the toxicity of CoCl_2_ in H9c2 cells, H9c2 cells were treated with different concentrations of CoCl_2_ for 24 or 48 h. MTT assay showed that treatment with CoCl_2_ at 600, 800, 1000, and 1200 µM significantly reduced the viability of H9c2 cells at both 24 h (*p * <  0.05, Fig. 1A) and 48 h (*p * <  0.05, Fig. 1B). TUNEL staining (Fig. 1C) and flow cytometry analyses (Fig. 1D) indicated that treatment with 800 µM CoCl_2_ for 48 h significantly promoted apoptosis of H9c2 cells (*p * <   0.05). In addition, CoCl_2_ treatment increased the levels of MAPK signaling members (p-JNK1/2, p-ERK1/2, and p-p38) and Notch1 signaling members (Notch1, Hes 1, and Jagged 1; *p*  <  0.05; Fig. 1E, F). These results suggested that CoCl_2_ could significantly inhibit proliferation, induce apoptosis, and activate MAPK and Notch1 signaling pathways in H9c2 cells.

**Fig. 2:**
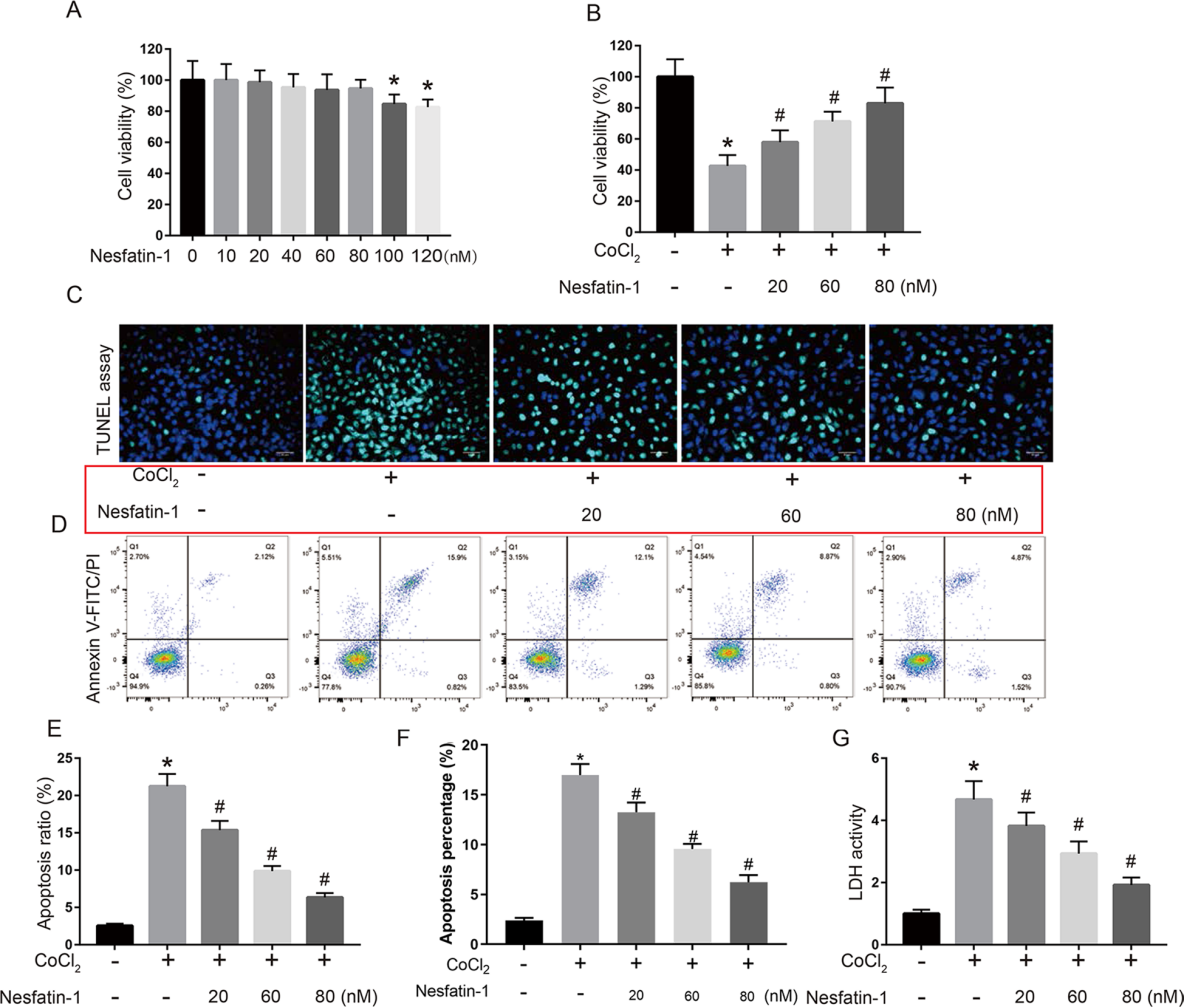
Nesfatin-1 protected H9c2 cells against CoCl_2_-induced cytotoxicity. **A** H9c2 cells were treated with nesfatin-1 (10, 20, 40, 60, 80, 100 and 120 nM) for 24 h, and cell viability was detected by MTT assay. **B**–**G** H9c2 cells were treated with 800 µM CoCl_2_ for 48 h before treatment with different concentrations of nesfatin-1 (20, 60 and 80 nM) for 24 h. **B** Cell viability was detected by MTT assay. Cell apoptosis was evaluated using TUNEL staining assay (**C**, **E**) and flow cytometry (**D**, **F**). **G** The release of LDH in the supernatant was detected using corresponding detection kit. Data are presented as mean ± SD. *p  <  0.05 vs. control group (0 µM CoCl_2_); #*p*  <  0.05 vs. CoCl_2_ alone group (800 µM CoCl_2_ and 0 nM nesfatin-1)

### Nesfatin-1 protected H9c2 cells against CoCl_2_-induced cytotoxicity

To explore whether nesfatin-1 is cytotoxic, H9c2 cells were treated with nesfatin-1 at 10, 20, 40, 60, 80, 100, and 120 nM for 24 h, and the cytotoxicity of nesfatin-1 against H9c2 cells were examined. MTT assay indicated that cell viability was not significantly changed after 10 to 80 nM nesfatin-1 treatment but significantly decreased after 100–120 nM nesfatin-1 treatment (*p*   <  0.05; Fig. 2A). To explore the effect of nesfatin-1 on CoCl_2_-induced cytotoxicity, H9c2 cells were treated with 800 µM CoCl_2_ for 48 h before incubation with 20, 60, and 80 nM nesfatin-1 for 24 h. MTT assay indicated that nesfatin-1 significantly reduced CoCl_2_-induced cytotoxicity and enhanced cell viability (*p*  <  0.05; Fig. 2B). In addition, TUNEL staining assay (Fig. 2C, E) and flow cytometry analysis (Fig. 2D, F) also showed that CoCl_2_-induced apoptosis in H9c2 cells was partially attenuated by nesfatin-1 treatment (*p * <  0.05), suggesting a potential cytoprotective effect of nesfatin-1 against CoCl_2_-induced apoptosis. Moreover, CoCl_2_ treatment significantly increased the release of LDH in H9c2 cells (*p * <  0.05), and nesfatin-1 effectively reverse this phenomenon (*p*  <  0.05; Fig. 2G). These results suggested that nesfatin-1 protected H9c2 cells against CoCl_2_-induced apoptosis.

### Nesfatin-1 protected H9c2 cells against CoCl_2_-induced ROS production

**Fig. 3:**
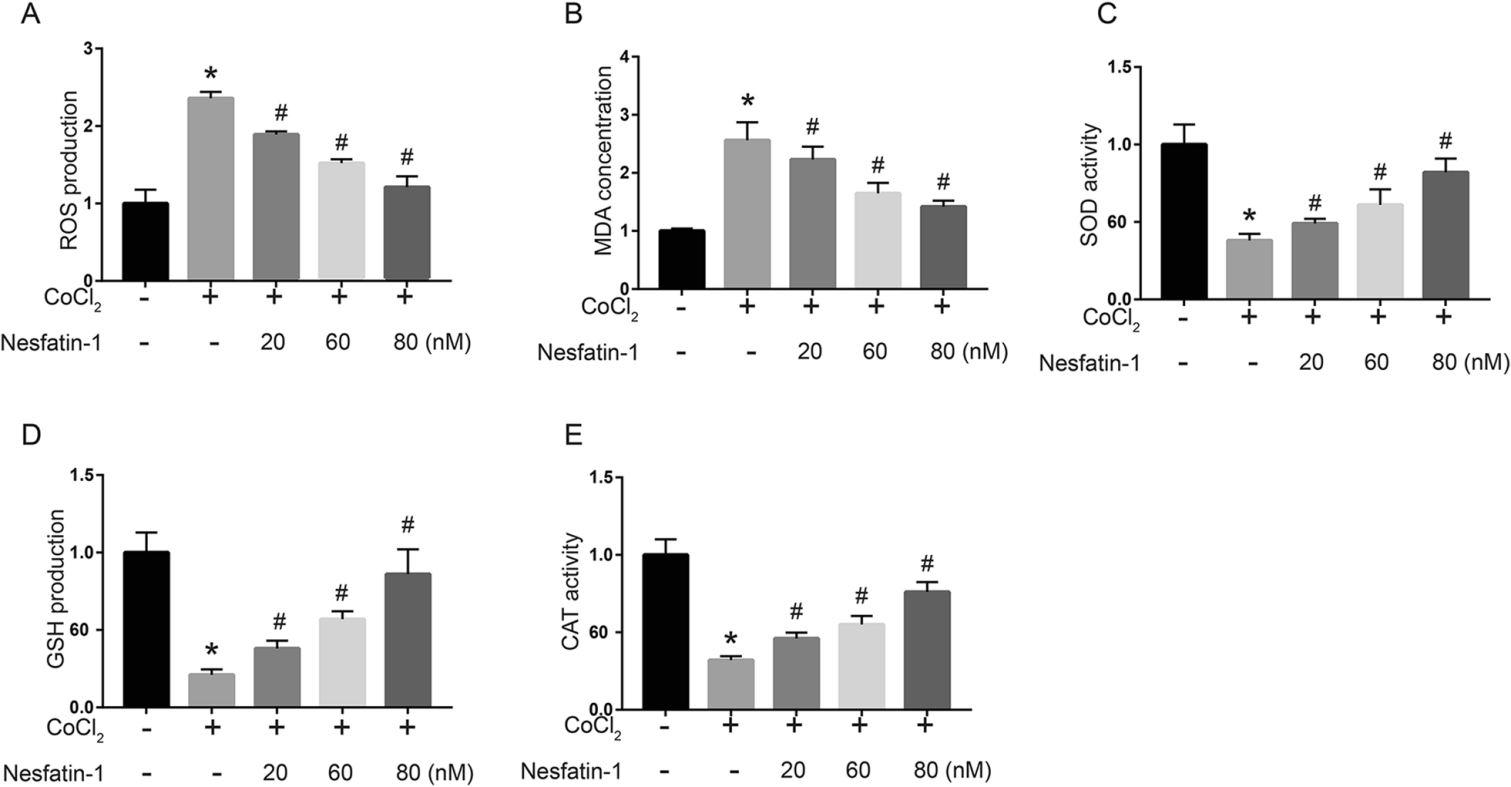
Nesfatin-1 protected H9c2 cells against CoCl_2_-induced ROS production. H9c2 cells were treated with 800 µM CoCl_2_ for 48 h, before treatment with nesfatin-1 (20, 60 and 80 nM) for 24 h. **A** The ROS level was detected using a fluorescence probe (DCFH-DA). The levels of released MDA (**B**), SOD (**C**), GSH (**D**) and CAT (**E**) in the supernatant were detected by corresponding detection kits. Data are presented as mean  ±  SD. *p  <  0.05 vs. control group (0 µM CoCl_2_); #*p*  <  0.05 vs. CoCl_2_ alone group (800 µM CoCl_2_ and 0 nM nesfatin-1)

Then we explored the effect of nesfatin-1 on CoCl_2_-induced ROS production. The intracellular ROS levels increased after CoCl_2_ treatment (*p * <  0.05), and nesfatin-1 significantly decreased intracellular ROS levels induced by CoCl_2_ (*p*  <  0.05), further confirming the antioxidant effect of nesfatin-1 (Fig. 3A). In addition, CoCl_2_ treatment significantly increased MDA concentration (*p*  <  0.05), while reduced SOD activity (*p * <  0.05), GSH production (*p * <  0.05), and CAT activity (*p*  <  0.05), and the oxidative abnormalities were obviously ameliorated by nesfatin-1 treatment (*p * <  0.05, Fig. 3B–E). These results revealed that nesfatin-1 effectively protected H9c2 cells against CoCl_2_-induced ROS production and oxidative abnormalities.

### Nesfatin-1 protected H9c2 cells against CoCl_2_-induced mitochondrial abnormalities

**Fig. 4:**
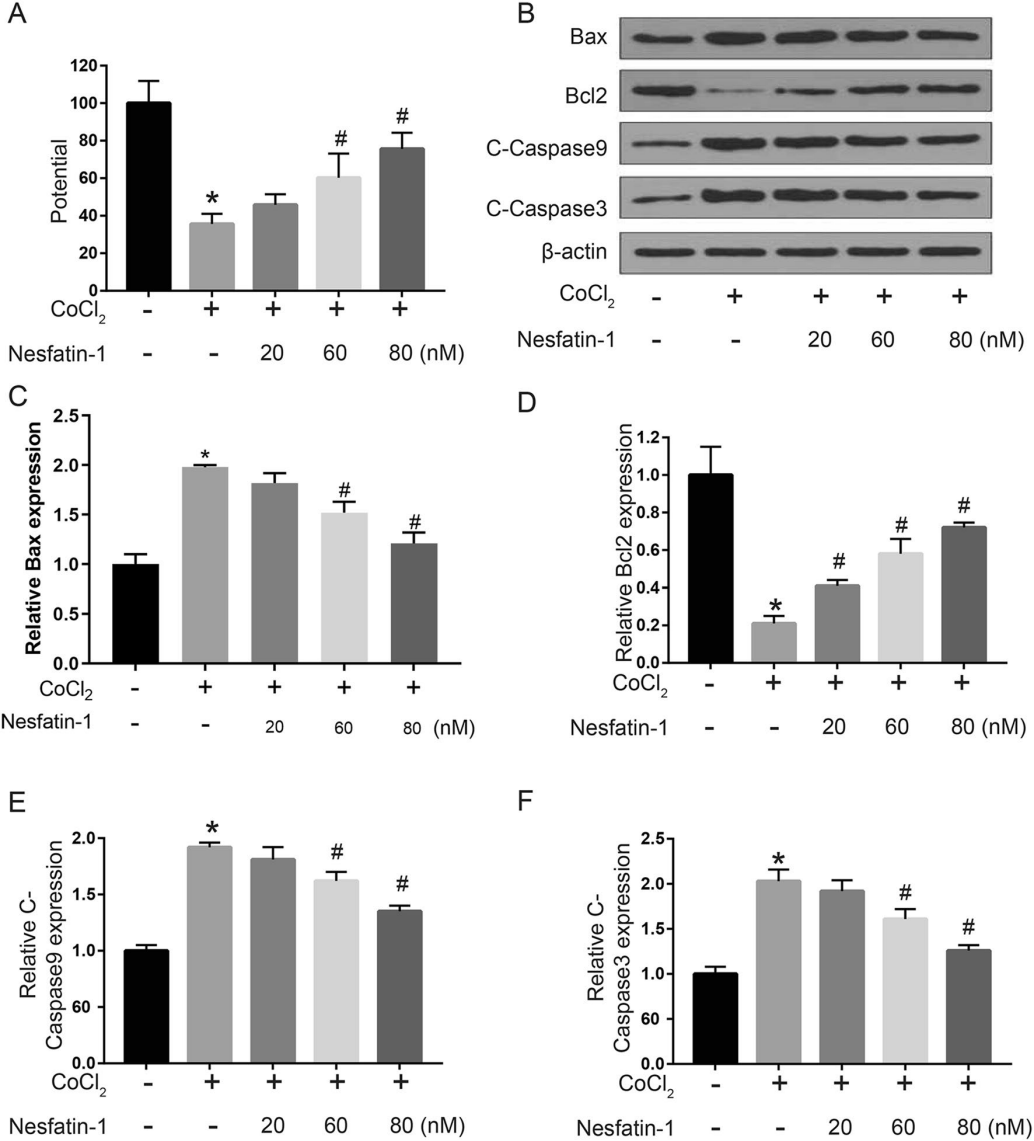
Nesfatin-1 protected H9c2 cells against CoCl_2_-induced mitochondrial abnormalities. H9c2 cells were treated with 800 µM CoCl_2_ for 48 h, before treatment with nesfatin-1 (20, 60 and 80 nM) for 24 h. **A** The MMP was detected using tetramethylrhodamine ethyl ester staining assay. **B**–**F** The expression of apoptosis-related proteins was detected by Western blot. Data are presented as mean  ±  SD. *p  <  0.05 vs. control group (0 µM CoCl_2_); #*p * <  0.05 vs. CoCl_2_ alone group (800 µM CoCl_2_ and 0 nM nesfatin-1)

Next, we explored the effect of nesfatin-1 on CoCl_2_-induced mitochondrial membrane potential (MMP) abnormalities. The results showed that CoCl_2_ treatment significantly decreased MMP of H9c2 cells (*p* < 0.05), while 60 and 80 nM rather than 20 nM nesfatin-1 obviously attenuated the loss of MMP induced by CoCl_2_ (*p*  <  0.05; Fig. 4A). Meanwhile, Western blot (Fig. 4B) indicated that CoCl_2_ treatment significantly increased Bax, c-caspase-9, and c-caspase-3 levels while downregulated Bcl-2 level in H9c2 cells (*p * <  0.05), and the abnormal expression of these apoptosis-related proteins was attenuated by 60 and 80 nM nesfatin-1 treatment (*p * <  0.05, Fig. 4C–F). These results demonstrated that nesfatin-1 could effectively protect H9c2 cells against CoCl_2_-induced mitochondrial abnormalities and apoptosis.

### Nesfatin-1 protected H9c2 cells against CoCl_2_-induced hypoxic injury by inhibiting MAPK and Notch1 signaling pathways

**Fig. 5:**
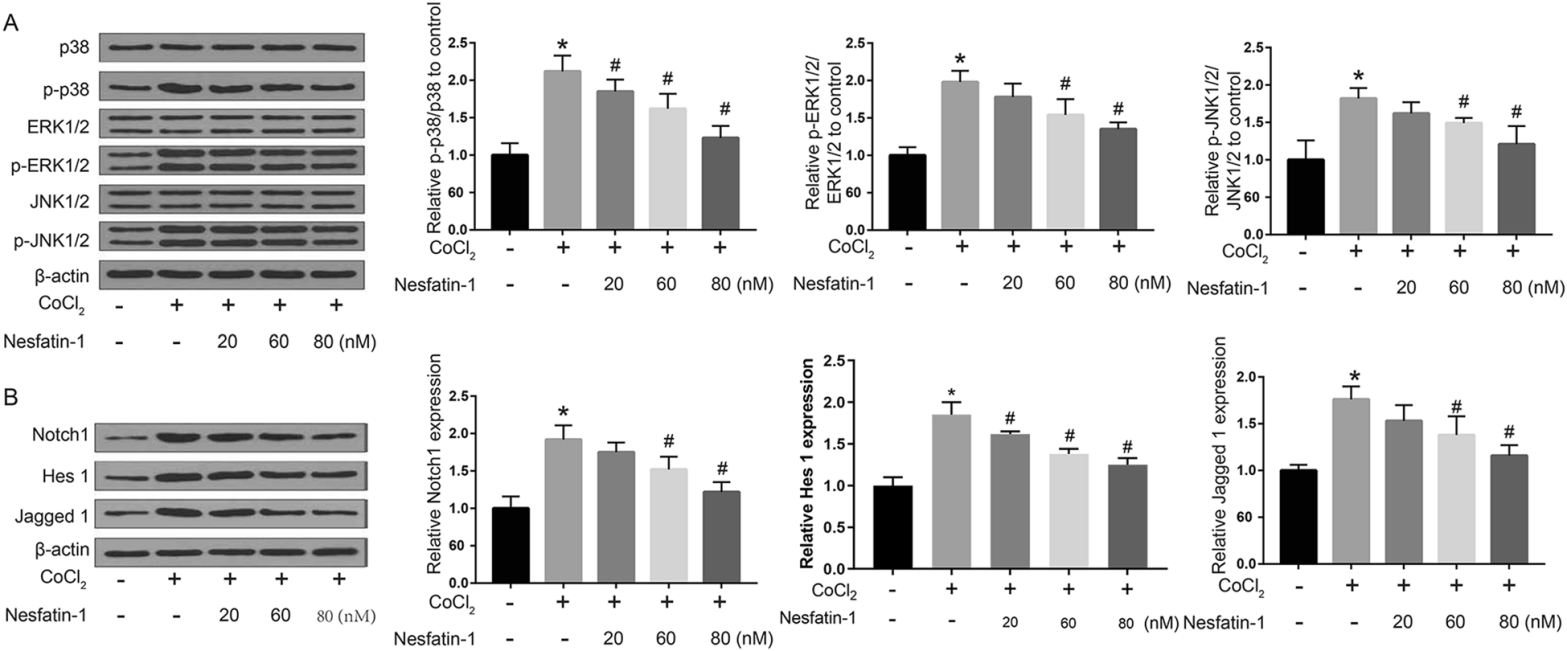
Nesfatin-1 protected H9c2 cells against CoCl_2_-induced hypoxic injury by inhibiting MAPK and Notch1 signaling pathways. H9c2 cells were treated with 800 µM CoCl_2_ for 48 h before treatment with nesfatin-1 (20, 60 and 80 nM) for 24 h. The expression of MAPK pathway members (**A**) and Notch1 pathway members (**B**) was detected by Western blot. Data are presented as mean  ±  SD. *p  <  0.05 vs. control group (0 µM CoCl_2_); #*p * <  0.05 vs. CoCl_2_ alone group (800 µM CoCl_2_ and 0 nM nesfatin-1)

Finally, we explored the effects of nesfatin-1 on CoCl_2_-induced MAPK and Notch1 signaling pathways. Western blot indicated that CoCl_2_ treatment significantly increased the levels of MAPK pathway members (p-JNK1/2, p-ERK1/2, and p-p38) and Notch1 pathway members (Notch1, Hes 1, and Jagged 1) (*p * <  0.05), while these elevations were attenuated by 60 and 80 nM nesfatin-1 treatment (*p * <  0.05; Fig. 5A, B). In addition, 20 nM Nesfatin-1 treatment alleviated CoCl_2_-induced upregulation of Hes 1 protein (*p * <  0.05; Fig. 5B). Overall, the results indicated that nesfatin-1 treatment protected H9c2 cells against CoCl_2_-induced hypoxic injury by inhibiting MAPK and Notch1 signaling pathways.

## Discussion

Hypoxia is a leading cause of irreversible organ damages, including the heart [24]. H9c2 cell line is widely used to explore the mechanism of hypoxia-induced cell damages [25]. CoCl_2_ is a well-known hypoxia-mimetic chemical reagent in vitro and in vivo [26, 27]. Consistently, treatment with CoCl_2_ at different concentrations decreased H9c2 viability and increased H9c2 apoptosis [28]. Importantly, the study revealed the significant protective effects of nesfatin-1 on CoCl_2_-induced cell injury. Moreover, our findings demonstrated that nesfatin-1 inhibits the activation of MAPK and Notch1 signaling pathways.

Due to the severe harm of hypoxia to the cardiomyocytes in the heart development, including decreased cell viability and enhanced cell apoptosis [29], several potential protective agents against hypoxia-induced injury in cardiomyocytes have been identified. For example, ginsenoside (Gin) Rg1 protects cardiomyocytes through upregulating the PI3K/AKT/mTOR pathway [30]. Astragaloside IV (AS-IV) protects cardiomyocytes by activating long non-coding RNA GAS5 mediated PI3K/mTOR signaling pathway [31]. Genistein (Gen) prevents cardiomyocytes from hypoxia-induced cell apoptosis by inhibiting the mitochondrial apoptotic pathway [32]. Ganoderic acid A (GAA) protects cardiomyocytes through upregulating miR-182-5p, downregulating PTEN, and activating PI3K/AKT signaling pathway [33]. Haemin attenuates hypoxia-induced cardiac injuries by reducing mitochondrial fission and cell apoptosis [34]. In addition, relaxin [35], sanggenon C [36], and trimetazidine [37] are potential agents against hypoxia-induced injury in cardiomyocytes. Although these agents exert certain protective effects on hypoxia-induced injury, some of them are toxic to other organs at high doses [38]. In this study, we found that 100–120 nM nesfatin-1 reduced the viability of H9c2 cells, indicating that high dose nesfatin-1 is toxic to cardiomyocytes. Hence, we selected the low dose nesfatin-1 to explore its effect and demonstrated that 20–80 nM nesfatin-1 could effectively attenuate CoCl_2_-induced injuries to H9c2 cells.

Oxidative stress is a key element leading to cell apoptosis in various pathological conditions [39]. ROS overproduction alters the mitochondrial structures, induces mitochondrial depolarization, decreases MMP, enhances pro-apoptotic molecule release, and causes cell apoptosis [40]. Previous studies have revealed that superoxide generation by the electron transport chain was elevated at hypoxia condition, resulting in ROS accumulation [41]. Here, CoCl_2_ was used to mimic the hypoxia condition, and CoCl_2_ treatment significantly reduced the viability and promoted apoptosis of H9c2 cells, stimulated ROS production, and led to cell injuries. Moreover, the CoCl_2_-induced damages to H9c2 cells were partially eliminated by the addition of nesfatin-1, suggesting that nesfatin-1 might be a potential protective agent against hypoxia-induced injuries.

Bcl-2, an anti-apoptotic factor, and Bax, a pro-apoptotic factor, are two crucial apoptosis-related proteins in the Bcl-2 family [42, 43]. In this study, we found that CoCl_2_ treatment significantly increased Bax, c-caspase-3, and c-caspase-9 levels while decreased the Bcl-2 level. Meanwhile, CoCl_2_ treatment-induced changes in apoptosis-related protein levels in H9c2 cells were reversed by nesfatin-1 treatment. Previous studies have demonstrated that ROS-dependent activation of MAPK and Notch1 signaling pathways was required for CoCl_2_-induced cell death in cardiomyocytes [32, 44]. Our results confirmed that the activation of MAPK and Notch1 signaling was associated with CoCl_2_-induced H9c2 cell apoptosis, and the activation of MAPK and Notch1 signaling pathways was markedly inhibited by nesfatin-1 treatment.

The role of Notch1 signaling in the hypoxia-injured cardiomyocytes is controversial. Boccalini et al. [45] reported that Notch1 expression was downregulated in hypoxia-induced H9c2 myocardial cells, and relaxin protected cardiomyocytes against hypoxia/reoxygenation injury by enhancing Notch-1 signaling. Hu et al. [46] and Yu et al. [47] have reported that activated Notch1 signaling attenuated hypoxia-induced injury in cardiomyocytes by reducing programmed cell death. These reports suggest that activated Notch-1 signaling might play a protective role in hypoxia-injured cardiomyocytes. However, Zhu et al. [48] has found that curcumin alleviates hypoxia/reoxygenation-induced cardiomyocyte injury by inhibiting Notch signaling, consistent with our findings in the present study. This discrepancy might be due to different mechanisms to induce hypoxia and needs to be further explored.

Our study has some limitations. First, our results were obtained by using rat embryonic cardiomyocyte H9c2 cell line and need to be confirmed in other cardiomyocyte cell lines. Second, nesfatin-1 concentration to exert its potential therapeutic or toxic effects may vary in different cells. Therefore, our findings need to be further confirmed in co-cultured cell models.

## Conclusions

In summary, we demonstrated that nesfatin-1 effectively protects cardiomyocytes from CoCl_2_-induced hypoxic injuries by inhibiting MAPK and Notch1 signaling pathways, suggesting that nesfatin-1 might be a potential therapeutic agent against hypoxic injuries to cardiomyocytes.

## Data Availability

The datasets produced and analyzed during the current study are available from the corresponding author on reasonable request.
